# Convergence: Lactosylceramide-Centric Signaling Pathways Induce Inflammation, Oxidative Stress, and Other Phenotypic Outcomes

**DOI:** 10.3390/ijms22041816

**Published:** 2021-02-12

**Authors:** Subroto Chatterjee, Amrita Balram, Wendy Li

**Affiliations:** Department of Pediatrics, Cardiology Division, Johns Hopkins University School of Medicine, Baltimore, MD 21287-3654, USA; abalram2@jhu.edu (A.B.); wli72@jhu.edu (W.L.)

**Keywords:** lactosylceramide, glycosphingolipids, cell proliferation, angiogenesis, inflammation, atherosclerosis

## Abstract

Lactosylceramide (LacCer), also known as CD17/CDw17, is a member of a large family of small molecular weight compounds known as glycosphingolipids. It plays a pivotal role in the biosynthesis of glycosphingolipids, primarily by way of serving as a precursor to the majority of its higher homolog sub-families such as gangliosides, sulfatides, fucosylated-glycosphingolipids and complex neutral glycosphingolipids—some of which confer “second-messenger” and receptor functions. LacCer is an integral component of the “lipid rafts,” serving as a conduit to transduce external stimuli into multiple phenotypes, which may contribute to mortality and morbidity in man and in mouse models of human disease. LacCer is synthesized by the action of LacCer synthase (β-1,4 galactosyltransferase), which transfers galactose from uridine diphosphate galactose (UDP-galactose) to glucosylceramide (GlcCer). The convergence of multiple physiologically relevant external stimuli/agonists—platelet-derived growth factor (PDGF), vascular endothelial growth factor (VEGF), stress, cigarette smoke/nicotine, tumor necrosis factor-α (TNF-α), and in particular, oxidized low-density lipoprotein (ox-LDL)—on β-1,4 galactosyltransferase results in its phosphorylation or activation, via a “turn-key” reaction, generating LacCer. This newly synthesized LacCer activates NADPH (nicotinamide adenine dihydrogen phosphate) oxidase to generate reactive oxygen species (ROS) and a highly “oxidative stress” environment, which trigger a cascade of signaling molecules and pathways and initiate diverse phenotypes like inflammation and atherosclerosis. For instance, LacCer activates an enzyme, cytosolic phospholipase A2 (cPLA2), which cleaves arachidonic acid from phosphatidylcholine. In turn, arachidonic acid serves as a precursor to eicosanoids and prostaglandin, which transduce a cascade of reactions leading to inflammation—a major phenotype underscoring the initiation and progression of several debilitating diseases such as atherosclerosis and cancer. Our aim here is to present an updated account of studies made in the field of LacCer metabolism and signaling using multiple animal models of human disease, human tissue, and cell-based studies. These advancements have led us to propose that previously unrelated phenotypes converge in a LacCer-centric manner. This LacCer synthase/LacCer-induced “oxidative stress” environment contributes to inflammation, atherosclerosis, skin conditions, hair greying, cardiovascular disease, and diabetes due to mitochondrial dysfunction. Thus, targeting LacCer synthase may well be the answer to remedy these pathologies.

## 1. Introduction

**Figure 1:**
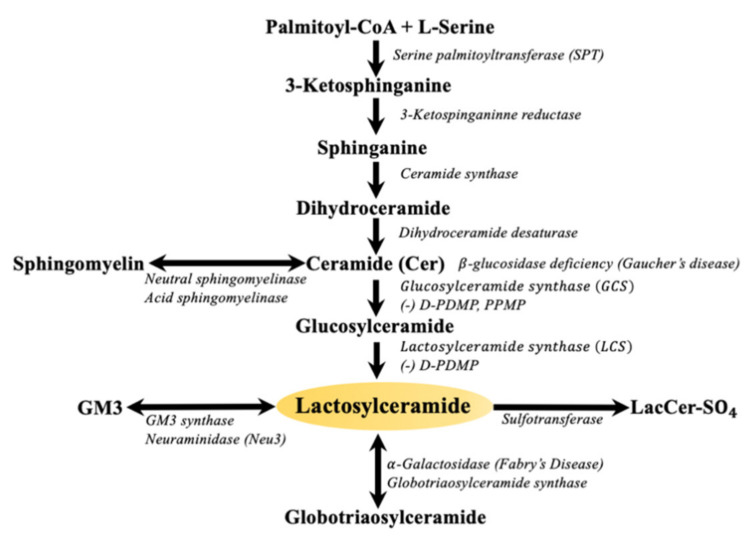
Biochemical pathways of lactosylceramide metabolism.

Glycosphingolipids (GSLs) are a family of small molecular weight molecules composed of fatty acids, sugars, and an amino acid. The biosynthesis of sphingolipids is initiated upon the condensation of its two fundamental constituents, palmitoyl-coenzyme A (palmityl-CoA) and L-serine, to form sphinganine. Next, the addition of another fatty acid leads to the formation of ceramide (Figure 1). Subsequently, sugars such as glucose or galactose are sequentially added via specific glycosyltransferases to ceramide giving rise to glucosylceramide, galactosylceramide, and about 300 complex GSLs [1]. The large body of literature gathered over the last three decades amply describe the important roles of sphingosine, sphingosine-1-phosphate (S1P) [2], and ceramide (Cer) [3] in regulating critical phenotypes such as cell proliferation, angiogenesis, and apoptosis. In fact, ceramide has become synonymous to apoptosis. Nevertheless, there are some critical or beneficial functions of ceramide such as serving as a barrier in the skin, which covers nearly the entire mammalian body. This is an area of ceramide biology that requires discussion. Thus, this review will attempt to bring this beneficial function of ceramide to the attention of readers. The next step in GSL synthesis involves the transfer of galactose from uridine diphosphate galactose (UDP-galactose) to GlcCer to form lactosylceramide (LacCer, Figure 1). LacCer can then be used to synthesize more complex GSLs, such as globosides, sulfatides, and gangliosides, in the Golgi apparatus [4].

In contrast to lipids used for structure and energy, bioactive lipids, which include sphingolipids, ceramide, sphingosine, and sphingosine-1-phosphate, are lipids that respond to specific stimuli and are parts of signaling pathways [3]. There is evidence that GlcCer and LacCer may be considered bioactive lipids. For instance, exogenous LacCer treatment is associated with increased cell adhesion, angiogenesis, reactive oxygen species, and inflammation independent of the above bioactive lipids [5]. The major focus of this review is to present a progress report on what we have learned about LacCer over the last several decades and how such knowledge can be applied in translational medicine. We aim to appreciate its potential role in health and disease as a target for therapy and in diagnosis by way of raising the predictive value of diseases. We will describe how glycosylation of Cer to LacCer can contribute to mitochondrial dysfunction in diabetes, skin health, hair loss/discoloration, and cardiac function. This special issue of glycosphingolipids will cover details on ceramide, sphingosine, and S1P among some lipids [2,3].

## 2. Lactosylceramide (LacCer) Synthesis via β-1,4 Galactosyltransferases V and VI and Physiological Implications

The enzyme lactosylceramide synthase (LacCer synthase) is a member of a large family of β-galactosyltransferases. This enzyme is localized in the trans-Golgi lumen where it has access to its substrate, GlcCer. Its function is to transfer galactose from a nucleotide sugar UDP-galactose to GlcCer to form LacCer. We first reported the purification of two LacCer synthases, encoded by the β-1,4 galactosyltransferase V (β-1,4GalT-V) and β-1,4 galactosyltransferase VI (β-1,4GalT-VI) genes, from the human kidney wherein they resolved into two protein bands: ~58 kDa and ~60 kDa [6]. Subsequently, β-1,4GalT-VI was cloned from rat brain [7]. Molecular studies showed a ~68% sequence homology between β-1,4GalT-V and β-1,4GalT-VI. Moreover, it was shown that β-1,4GalT-V was constitutively expressed in various human embryonic tissues as well as adult tissues, while β-1,4GalT-VI was specifically expressed in human brain [8]. The physiological effects of LacCer synthases encoded by the β-1,4GalT-V and β-1,4GalT-VI genes play a crucial role in neuronal generation as well as formation of myelin in mice [9]. Herein, mice with double knockout of these genes in the central nervous system did not produce gangliosides like monosialotetrahexosylceramide (GM1) thus lacking interaction with laminin and resulting in immature neurons and perineuronal cells in the cerebral cortices. Mouse extra-embryonic development required the β-1,4GalT-V gene during early embryogenesis, and its disruption could be lethal [10]. However, soon, a controversy surfaced in the literature claiming that β-1,4GalT-V is an N-glycan, O-glycan synthase and not a LacCer synthase [11]. Using a mutant Chinese hamster ovary (CHO) cell line (gift from Dr. Pamela Stanley), which exclusively expressed β-1,4GalT-V, we showed that this enzyme could synthesize LacCer [12]. 

**Figure 2:**
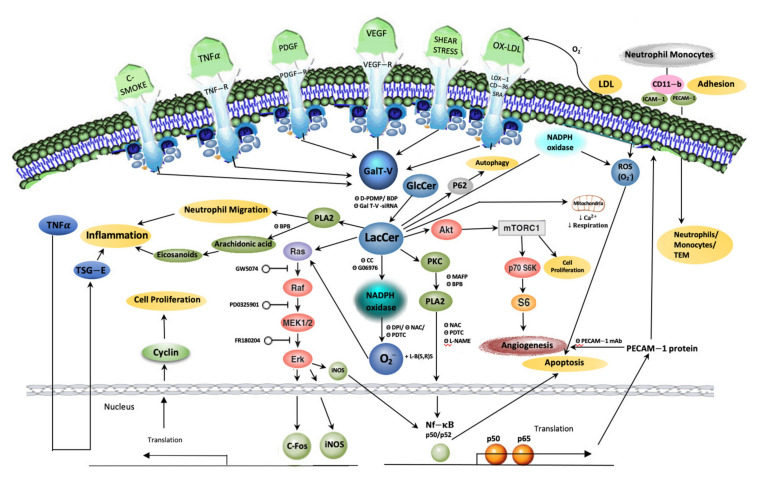
Schema of lactosylceramide-centric signaling pathways in the regulation of multiple phenotypes. Diverse agonists converge upon lactosylceramide (LacCer) synthase to generate LacCer. In turn, LacCer creates an “oxidative stress” environment by way of generating reactive oxygen species (ROS)/superoxides. LacCer also activates cytosolic phospholipase A2 (cPLA2) to generate arachidonic acid, a precursor to prostaglandins. Collectively, induction of these two signaling pathways contribute to diverse phenotypes: inflammation, cell proliferation, migration/infiltration, adhesion, angiogenesis apoptosis, autophagy, and mitochondrial dysfunction.

Platelet-derived growth factor (PDGF) is secreted by platelets and other vascular cells in response to changes in hemodynamic conditions. It contributes toward the proliferation of arterial smooth muscle cells as well as their migration from the tunica media (the middle of the arterial wall) to the tunica intima (the inner layer of the arterial wall), thus contributing to atheroma formation. In mutant CHO cells, PDGF stimulated the activity of β-1,4GalT-V to generate LacCer and contributed to cell proliferation via recruiting an elaborate cell signaling pathway involving reactive oxygen species (ROS) generation, kinase cascade activation, Akt-1 [12] and mammalian target of rapamycin (mTORC1, an established master-regulator of growth and proliferation) [13,14,15] (Figure 2).

A critical physiologically relevant observation was made when it was revealed that β-1,4GalT-V is highly enriched in vascular tissue—in particular, in the single layer of endothelial cells which constitute the surface of blood capillaries and are directly exposed to circulating blood and its elements. This topology of β-1,4GalT-V may directly impact the vascular biology and functions of the cardiovascular system (see below) and lungs via regulating critical phenotypes including cell–cell adhesion, apoptosis, angiogenesis, and autophagy. 

## 3. Lactosylceramide Localization

GSLs make up a component of lipid rafts of the plasma membrane and can serve as binding sites or pattern-recognition receptors for various extracellular ligands [16]. LacCer-containing lipid rafts on plasma and granular membranes of human neutrophils can bind to specific carbohydrates displayed by pathogens such as *Mycobacteria* and *Candida.* This fosters an innate immune response in the host cell by activating Src family kinases and a series of processes including chemotaxis and phagocytosis. Human dendritic cells can also be activated by β-Glucan from *Pneumocystis carinii*, leading to the release of interleukin-23 (IL-23) from dendritic cells. Furthermore, these LacCer-rich lipid rafts utilize a mediator, αMβ2-integrin, to help signal inflammation and phagocytosis [16]. 

Immunohistochemistry using a monoclonal mouse anti-human CD17 antibody against LacCer [17] revealed that LacCer is mostly enriched within the cytoplasmic vesicles in proximal tubular cells from familial hypercholesterolemic (FH) homozygous patients. Similar results were found in patients with human polycystic kidney disease [18,19]. We speculate that these LacCer-carrying vesicles bud out of the Golgi apparatus and represent the “storage form” of LacCer. On the other hand, surface plasmon resonance analysis revealed that T5A7, a novel monoclonal antibody against LacCer, specifically binds to the LacCer localized on the cell surfaces of mouse and human neutrophils [20]. The binding of LacCer to neutrophils was accompanied with neutrophil migration and contributed to phagocytosis. This phenotype was facilitated by Lyn, a member of the Src family kinases, as well as phosphoinositide 3 (PI-3)-kinases [20]. These studies also showed the binding of T5A7 to a lipid monolayer consisting of LacCer and major lipid cell membrane components such as cholesterol, sphingomyelin, and phospholipids. Huly, another monoclonal antibody against LacCer, was unable to recognize cell-surface-associated LacCer but it did recognize the bulk of cellular LacCer localized within the cytoplasmic vesicles. This duality in recognizing the same antigen is a fascinating observation which needs to be examined further, and its physiological significance needs to be explained [21]. 

In blood, LacCer is associated with low-density lipoproteins (LDL) and high-density lipoproteins (HDL) and delivered to peripheral tissues. A significant amount of β-1,4GalT-V has also been found to be associated with the cytoplasm in human colorectal cancer tissue [15]. However, a small amount of β-1,4GalT-V is associated with the cell membrane, consequently generating a small amount of LacCer within this organelle [15,17,22]. LacCer has been shown to serve as a receptor for a wide range of bacteria as well as the yeast *Candida albicans* implicated in urinary tract infections [23]. Other studies showed that LacCer interacts with 1,3-β-glucan in human leukocyte membrane [24], inducing several pro-inflammatory cytokines: TNF-α and macrophage inflammatory protein 2 (MIP-2), which enhances oxidative burst and anti-microbial function of leukocytes [25]. Such functional activities of β-glucans are useful in the prevention of cervical cancer [25]. Also, exogenously supplied LacCer can induce cell proliferation, angiogenesis and cell migration [5]. All this evidence may suggest the involvement of a receptor-mediated process, but this needs to be examined further.

The interactions between GSLs and pathogen-associated carbohydrates can potentially be used to better understand certain immune diseases. For instance, capsular polysaccharide from *Streptococcus suis* prevents LacCer-induced phagocytosis of the pathogen by macrophages [16]. Similarly, *Mycobacteria* evades destruction by the host immune system by interfering with the innate immune functions of the GSL-rich lipid rafts. In sum, GSLs play an important role in the dynamics and integrity of the cell membrane [26]. Since LacCer is highly enriched in lipid rafts, it may well serve as “gateway” for interactions with various physiologically relevant molecules, enabling multiple cellular responses [5,27].

## 4. Lactosylceramide Synthesis via Neuraminidase, α-Galactosidase, and Sphingomyelinases

Human liver contains a β-1,4-galactosidase shown to cleave the terminal β-1,4-galactose residue in LacCer and GM1 ganglioside [28]. This enzyme cleaves LacCer to form GlcCer as we observed in our metabolic experiment using [3H]-LacCer adsorbed onto LDL in a FH homozygous patient. 

LacCer can be generated by the action of several different enzymes. Studies show that a cell-membrane-bound neuraminidase-3 (Neu3) can cleave the terminal neuraminic acid residue in monosialosyldihexosylceramide (GM3) and disialosyldihexoyslceramide (GD3) to generate LacCer [29]. The generation of LacCer via this reaction has been associated with several types of human cancers [30]. Neu3 has been reported to inhibit apoptosis and is, accordingly, upregulated in several cancers including colon cancer progression [31]. There were higher levels of Neu3 messenger RNA (mRNA) in the epithelial part of adenocarcinoma than in the nearby normal tissue. Neuraminidase-1 (Neu1)-catalyzed generation of LacCer has also been implicated in inducing elastin-derived peptides to decrease adipocyte differentiation [32]. Herein, studies show that the composition and structure of the extracellular matrix (ECM) can be altered by proteases such as matrix metalloproteinases (MMPs) released by adipocytes and macrophages. In damaged or old tissues, elastin, a major ECM fiber that allows tissues to stretch, is degraded by MMPs into elastin-derived peptides. These peptides impact atherosclerosis, thrombosis, and metabolic diseases by activating the elastin receptor complex. The cytosolic C-terminal of this complex contains a neuraminidase-1 (Neu1) catalytic subunit. Although the function of Neu1 is not yet elucidated, it may cleave sialic acid residues from GM3 ganglioside to generate LacCer, which is proposed to be a second messenger of the activated elastin receptor complex. 

LacCer generation can also involve a monovalent cationophore called monensin. It is known to disrupt the Golgi apparatus, thus altering the secretion of newly synthesized glycoproteins and recycling of LDL receptors [33,34,35]. In neuronal cells, human fibroblasts, and human kidney proximal tubular cells, monensin increased the levels of GlcCer and LacCer [36,37,38]. Detailed enzymatic studies suggested that monenisn-induced increase in LacCer level of human kidney proximal tubular cells was due to increased α-galactosidase activity, therefore catabolizing globotriosylceramide (GbOse3Cer) to LacCer. These studies also showed that monensin decreased LacCer synthase activity by disrupting the Golgi apparatus.

Lastly, LacCer can be generated in a series of steps involving the action of neutral sphingomyelinase (N-SMase) and acid sphingomyelinase. Sphingomyelinases constitute a family of phospholipases which cleave a phospholipid called sphingomyelin into phosphocholine and ceramide (Figure 1). Several forms of these enzymes have been shown to be associated with the cell membrane, endoplasmic reticulum, lysosome, mitochondria, and nucleus. The ceramide generated by sphingomyelinase action is subsequently converted to various bioactive sphingolipids including LacCer [39]. Studies suggest that LacCer, but not ceramide, partakes in vital mitochondrial functions: respiration, Ca^2+^ retention, apoptosis, and perhaps in necrosis (see below).

## 5. Role of the Low-Density Lipoprotein (LDL) Receptor in Determining the Metabolic Fate of LacCer

**(i)**  In vitro metabolism of LacCer

A significant amount of LacCer is synthesized in the liver and presumably assembled onto very-low-density lipoprotein (VLDL) and LDL for delivery to various extra-hepatic tissues. Studies using normal human kidney proximal tubular cells and LDL-receptor-negative homozygous familial hypercholesterolemic (FH) subjects showed that LDL dose-dependently reduced the synthesis of LacCer in normal cells. In contrast, it stimulated the synthesis of LacCer in LDL-receptor-deficient mutant cells. In addition, when the lysine residues in LDL were reductively methylated, it rendered a loss of LDL’s ability to decrease LacCer synthesis in normal cells [40] with functional LDL receptors.

A mechanistic explanation of this observation was revealed with the finding that LDL, but not HDL, suppressed LacCer synthesis in normal human kidney proximal tubular cells. In contrast, reductive methylation of lysine residues in LDL increased the activity of LacCer synthase several-fold. Since the lysine residues in LDL are essential for its binding to LDL receptors located on the surfaces of mammalian cells, methylation deprived such recognition. Thus, methylated LDL entered the cells via an LDL-receptor-independent pathway/scavenger pathway. These observations were recapitulated in additional cell-based assays using normal human skin fibroblasts, CHO cells, and bovine smooth muscle cells [40]. Inhibition was much stronger in semi-confluent cells, which expressed an increased number of LDL receptors compared to high-density cell cultures [41]. Collectively, these studies showed that LDL taken up via the LDL-receptor pathway suppressed LacCer synthesis. In contrast, LDL taken up via LDL-receptor-independent pathway or “scavenger pathway” stimulated LacCer synthesis.

This observation meant that using cells from patients with atherosclerosis also had important implications in human renal cancer [42]. For example, it was reported that human renal cancer cells are LDL-receptor-deficient. Consequently, feeding these cells with LDL dose-dependently increased the activity of LacCer synthase and raised the mass of LacCer [42]. Further molecular signaling studies revealed that LacCer functioned independently as a mitogenic agent (see below).

Metabolic studies in normal human kidney proximal tubular cells showed that [3H]-LacCer tagged onto LDL was taken up via the LDL receptors [40]. Subsequently, it was transported to the lysosome within 30 min and catabolized to GlcCer. LacCer also served as a precursor for its higher homologs: globotriosylceramide (GbOse3Cer) and ganglioside GM3. In contrast, in renal cells from LDL-receptor-negative, homozygous FH patients, [3H]-LacCer tagged onto LDL was not rapidly metabolized. Rather, it was stored in large cytoplasmic vesicles and eventually metabolized [40].

**(ii)**  In vivo metabolism of LacCer in man

In vivo uptake and metabolism of [3H]-LacCer adsorbed onto [125I]-tagged LDL was examined in FH homozygous patients [43]. The bulk of [3H]-LacCer radioactivity injected into blood was rapidly removed from the plasma within 4 h of injection. Subsequently, the level of [3H]-LacCer associated with the patient plasma declined slowly, while [125-I] associated with the LDL had a half-life of 6 days. In the liver, radioactivity was associated exclusively with GlcCer. In contrast, in the kidney, [3H]-LacCer radioactivity was the predominant form of radioactivity; a small percentage of radioactivity was associated with GlcCer and GbOse3Cer. Furthermore, intact renal proximal tubular cells shed in the urine of these patients contained the vast majority of [3H]-LacCer radioactivity. This suggests that a significant proportion of blood LacCer is removed by the excretory system. This tenet was confirmed with the finding that urinary cells of FH patients shed ~10 times as much LacCer compared with that of normal subjects [44]. Electron microscopy of kidney tissue sections revealed the presence of numerous osmium-tetroxide-positive cytoplasmic vesicles, suggestive of lipid accumulation [17]. Thus, these in vivo studies in LDL-receptor-negative FH patients underscore the importance of the LDL-receptor-independent pathway in the uptake, metabolism, and storage of LacCer in the kidney and the shedding of LacCer adsorbed onto LDL. Moreover, these findings underscore the importance of urinary sediment cells as a non-invasive source of patient sample to study GSL metabolism and excretion in metabolic, cardiovascular, and inherited diseases.

## 6. Lactosylceramide Metabolomics and Cardiovascular Function

Advances in mass spectrometry (MS) have greatly contributed to rapid progress in our attempts to conduct lipid metabolomics and studies analyzing sphingolipids in experimental animals [13,15,45,46] and in a large number of human samples from diabetic patients to patients with cardiovascular disease. These studies supported a correlation between increased GSL levels and alterations in cardiovascular function and CVD risk stratification. For example, two recent reports suggested that there is a strong association between LacCer level and arterial stiffness (AS). AS indicates a loss in arterial elasticity, a mechanical property of the artery. First, we measured the arterial level of LacCer by MS and pulse-wave velocity (PWV, a measure of AS determined by high-resolution Doppler spectrum analyzer) in a mouse model of atherosclerosis (Apolipoprotein E deficient, *ApoE^−/−^* mice) fed a high fat and high cholesterol diet [46]. In the mice from age 20 weeks to 36 weeks, there was a high positive correlation (r = 0.96) between the level of LacCer and PWV, suggesting increased arterial stiffness and vascular dysfunction. When a parallel group of mice were fed D-threo-1-phenyl-2-decanoylamino-3-morpholino-1-propanol (D-PDMP), a GSL synthesis inhibitor (orally by gavage daily), it reduced both the level of LacCer and PWV [46]. 

Studies with *ApoE*^−/−^ mice were further substantiated in humans. In a human group of overweight middle-aged subjects and subjects progressing to impaired fasting glucose, there was a high correlation (r = 0.75) between PWV and LacCer level [47]. In another metabolomics study of several thousand type-2 diabetic patients, increased levels of serum GlcCer and LacCer levels markedly enhanced the predictive value of cardiovascular disease progression events and mortality when combined with other established risk factors [48]. Moreover, exploratory analysis of large-scale lipidome in large cohorts revealed that the plasma GSL signature was strongly associated with cardiovascular disease (CVD) outcomes: non-fatal CVD events were similar to GSL values preceding CVD death [49]. In other words, the increased plasma level of GSLs observed prior to a CVD event was a harbinger of future CVD events and death. Thus, analysis of discriminant GSLs and other sphingolipids may well accelerate clinical decision making in patients with CVD and related diseases—diabetes, obesity, cerebro-vascular disease, and possibly Alzheimer’s disease.

## 7. LacCer in Cell Proliferation: Recruitment of Reactive Oxygen Species, a Kinase Cascade, Proto-Oncogene c-Fos, and Proliferating Cell Nuclear Antigen 

Arterial smooth muscle cells form the bulk of cells in blood vessels. However, hemodynamic factors and growth factors, like PDGF, can stimulate their proliferation in the tunica media, as well as their migration to the tunica intima, to form atherosclerotic plaques. Thus, arterial smooth muscle cell proliferation is considered a “hallmark” in pathophysiology in atherosclerosis. However, the role of GSLs in proliferation was not known until comparative studies using arterial smooth muscle cells and 15 different sphingolipids were undertaken [50]. These studies revealed that gangliosides inhibited cell proliferation, sulfatides imparted no effect, and several neutral GSLs stimulated proliferation. In particular, LacCer exerted the highest increase, ~5 fold, in cell proliferation. The relevance of this observation to human disease was made when the levels of GlcCer and LacCer were found to be markedly elevated in atherosclerotic plaque and calcified plaque compared to unaffected aorta from the FH patients [51]. Further studies elaborated that LacCer stimulated the loading of GTP on Ras, followed by phosphorylation of Raf, mitogen-activated protein kinase 1/2 (MEK 1/2), and Erk, also known as mitogen-activated protein kinase (MAPK). Further downstream, LacCer, but not ceramide or GlcCer, stimulated the phosphorylation of MAPK p44 in human and rabbit arterial smooth muscle cells [52,53]. In particular, LacCer phosphorylated the tryptophan, threonine and serine residues in MAPK p44 in a descending order, suggesting a mitogenic role in atherosclerosis. This observation was recapitulated in other human proliferative diseases such as renal cancer and colorectal cancer (see below).

It is known that induced expression of proto-oncogenes—c-Jun, c-Myc and c-Fos—is an early response to various growth stimuli [54]. These proto-oncogenes encode several factors which bind to DNA in the nucleus to induce cell proliferation. In human arterial cells, LacCer specifically induced the expression of c-Fos to simulate cell proliferation (Figure 2).

The role of ROS in LacCer-mediated arterial smooth muscle cell proliferation was established when it was observed that ROS activated a “redox stress” signaling molecule, NADPH oxidase (but not NADH oxidase) [55]. A detailed mechanistic/molecular explanation how LacCer increases NADPH oxidase activity is not known. However, it has been reported that this process requires the migration and assembly of various cytosolic and membrane components of NADPH oxidase including p47 phox and p67 phox [56]. Other studies showed that NADPH oxidase activation via activation of Rac2 by exchange of GDP from GTP as well as phosphorylation and assembly of p47 phox with other components of NADPH oxidase [57]. These studies revealed a high order of specificity for LacCer to generate ROS as other GSLs (Cer, GlcCer, and GbOse3Cer) failed to generate ROS. Other studies have shown that c-Fos gene expression is altered due to changes in “redox state” [58].

Proliferating-cell-nuclear-antigen (PCNA) is a DNA clamp that facilitates the holding/processing of DNA polymerase to DNA in eukaryotic cells and plays an essential role in the DNA repair and synthesis phase of the cell cycle [59]. A marked accumulation of PCNA is noted in the nucleolus late in the G1 phase and early S phase of the cell cycle. In arterial smooth muscle cells, treatment with LacCer induced the expression of PCNA/cyclin as judged by Western immunoblot assays [60]. Thus, induction of an immediate early gene, such as LacCer synthase, and/or LacCer itself triggers an elaborate signaling cascade yielding c-Fos induction and the subsequent expression of PCNA/cyclins, ultimately increasing cell proliferation.

## 8. Oxidized LDL Recruits LacCer Synthase/LacCer to Induce Cell Proliferation and Atherosclerosis

The key steps in atherosclerosis are considered to be: an injury to the vascular endothelium, the uptake of LDL and its subsequent oxidation to oxidized LDL (ox-LDL), expression of cell adhesion molecules allowing the trans-endothelial migration (TEM) (Figure 2) of circulating monocytes/neutrophils, conversion of monocytes to macrophages which express receptors to take up ox-LDL, transformation of macrophages into lipid-laden foam cells, and the proliferation and migration of arterial smooth muscle cells [61,62]. A major breakthrough in LacCer research occurred when a study reported that oxidized LDL–a physiologically relevant molecule present in serum and human atherosclerotic plaques as well as in experimental animal models of human atherosclerosis–could dose-dependently activate LacCer synthase. This is of importance, since ox-LDL level is a stronger predictor of acute coronary heart disease than LDL [63,64]. However, in human arterial smooth muscle cells, LacCer synthase activation by ox-LDL was not associated with a gain in gene expression. Rather, it was due to rapid phosphorylation of the serine, threonine, and tryptophan residues in LacCer synthase [65]. A tyrosine kinase activator, phorbol-myristyl acetate, increased the activity of LacCer synthase whereas, tryphostin, an inhibitor of tyrosine phosphorylation, inhibited activity. However, vanadate, a protein phosphatase inhibitor, did not affect ox-LDL–induced increase in LacCer synthase activity. Thus, phosphorylation of the tyrosine, threonine and serine residues in LacCer synthase could be a potential mechanism by which ox-LDL increases LacCer synthase activity. 

Detailed follow-up studies on individual ox-LDL components revealed that oxidized phospholipids, such as 1-palmitoyl-2-(5-oxovaleroyl)-sn-glycero-3-phosphatidylcholine (POVPC), are present in minimally ox-LDL and that ox-LDL specifically mediated the activation of LacCer synthase activity [66]. In minimally oxidized LDL, a majority of lipid species are oxidized. In comparison, both the lipid and protein components are oxidized in ox-LDL [50]. Furthermore, ox-LDL induced arterial smooth muscle cell proliferation by activating an oxygen-sensitive signaling pathway (Figure 2). Activation of LacCer synthase was essential, as treatment with GSL synthesis inhibitor D-PDMP mitigated POVPC as well as ox-LDL–induced cell proliferation [66]. Conversely, treatment with L-threo-1-phenyl-2-decanoylamino-3-morpholino-1-propanol (L-PDMP), which activates LacCer synthase, stimulated cell proliferation. The newly generated LacCer followed the molecular signaling pathway components, p21 Ras, the kinase cascade, and c-Fos, contributing to increased expression of proliferating cell nuclear antigen/cyclin [60] and ultimately cell proliferation (Figure 2).

## 9. LacCer Induces LDL Oxidation in a Cyclical Fashion: Effects of Feeding a Western Diet in *ApoE^−/−^* Mice

Besides serving as a conduit in ox-LDL/POVPC–induced cell proliferation, LacCer may also play an important role in the oxidation of LDL. In an experimental animal model of atherosclerosis (*ApoE^−/−^* mice), feeding a western diet (2% high fat and 1.25% high cholesterol) increased the serum level of ox-LDL as well as the activities of GlcCer synthase and LacCer synthase [46]. However, as the time of feeding the western diet was raised from 20 weeks of age in *ApoE^−/−^* mice to 36 weeks, a ~10-fold increase in the activity of LacCer synthase was noted and confirmed by western immunoblot assays. In contrast, the activity of GlcCer synthase did not change significantly. Thus, aging alone raises the activity and mass of LacCer synthase, accompanied with a rise in LacCer level and ROS, and this effect is exacerbated further with a western diet. Additionally, studies have found that LacCer accumulates in the mouse kidney and may contribute to senescence, a process by which cells stop dividing [67].

**Figure 3:**
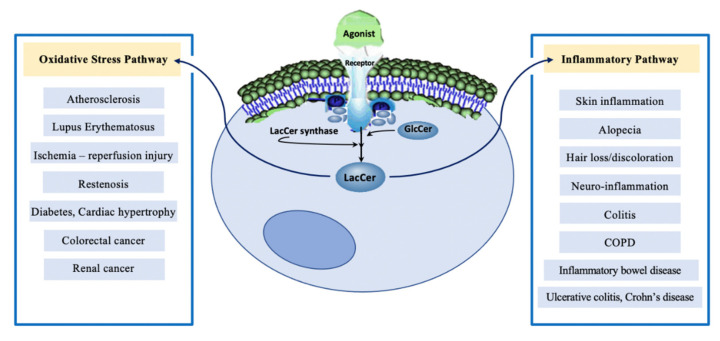
Schema of lactosylceramide in the regulation of multiple pathologies. Diverse agonists activate LacCer synthase to generate LacCer. LacCer induces two main pathways-oxidative stress and inflammatory. The oxidative stress pathway contributes to several pathologies: atherosclerosis, lupus erythematosus, ischemia, restenosis, diabetes, colorectal cancer, and renal cancer. The inflammatory pathway contributes to skin inflammation, alopecia, hair loss/discoloration, neuro-inflammation, colitis, chronic obstructive pulmonary disease (COPD), inflammatory bowel disease, ulcerative colitis (UC), and Crohn’s disease (CD).

On the other hand, feeding a western diet decreased the expression of the superoxide dismutase (SOD) I and II genes which encode for enzymes that convert superoxides, harmful byproducts of oxygen metabolism, into oxygen and hydrogen peroxide [68]. The excess generation of ROS due to the rise in LacCer level and reduction in the salvage of ROS (due to decreased SOD activity) may collectively contribute to increased LDL oxidation. Conversely, treatment with D-PDMP dose-dependently decreased the activity of LacCer synthase and LacCer mass, and increased SOD-I, catalase, hypoxia-inducible factor 1-alpha (HIF-1A), and SOD-II mRNA—the latter a mitochondrial enzyme (see below) [46]. Thus, a western diet and aging raises the levels of LacCer synthase and LacCer. In turn, LacCer generated as a consequence produces more ROS, which may escape the cell membrane and oxidize LDL. Such a cyclical reaction may explain a close association between increased LacCer levels, ROS, and LDL oxidation in experimental atherosclerotic and aging mice. The relevance of this finding in mice to man was recapitulated recently in patients with systemic lupus erythematosus wherein a strong association between LacCer synthase and ox-LDL level (*p* < 0.0001) was noted [69] (Figure 3). Since neutrophils carry the highest load of LacCer compared to other blood cells, a differential production of ROS by neutrophils in Lupus patients may help explain extensive oxidative damage including LDL oxidation [70].

## 10. LacCer in Mitochondrial Function, Apoptosis, and Necrosis

Mitochondria-associated endoplasmic reticulum (ER) membranes (MAMs) are structures that link the ER and the mitochondria and allow for cross-communication between the two organelles [4]. MAMs have similar structures as lipid rafts of plasma membrane and function as sites for membrane trafficking, lipid synthesis, calcium signaling, autophagy, apoptosis, and other mitochondrial processes. The accumulation of certain GSLs in MAMs is associated with disease states. For instance, the lysosomal storage disorder GM1-gangliosidosis results from the accumulation of GM1, which can interfere with calcium signaling and initiate neuronal apoptosis [4]. In mice models with streptozotocin-induced type 1 diabetes, LacCer was the main sphingolipid that decreased electron transport chain function and calcium retention capacity in the heart. In a novel study, the overexpression of UDP-glucose ceramide glucosyltransferase (UGCG) in breast cancer cells resulted in higher levels of both glucosylceramide and lactosylceramide in the ER and mitochondria [71]. This accumulation of GSLs led to increased oxidative phosphorylation, ROS levels, and mitochondrial turnover. The presence of UGCG and LacCer synthase in MAMs may suggest that GSLs are directly formed from the ceramide pool generated in the mitochondria. 

While the incubation of human arterial smooth muscle cells with low concentrations of ox-LDL induced cell proliferation, high doses of ox-LDL caused cell death [72]. Subsequent studies revealed that ox-LDL stimulated apoptosis via the activation of a neutral sphingomyelinase and the consequent generation of ceramide in human arterial smooth muscle cells [73]. Ox-LDL induced apoptosis not only by the mitochondrial apoptotic pathway, but also by the death-receptor (Fas/FasL) apoptotic pathway. Thus, activating the caspase cascade involves caspase-9 followed by cytochrome-c release from the mitochondria and typical apoptotic morphological features, such as DNA fragmentation and cellular membrane blebbing. On the other hand, ox-LDL triggers necrosis via a calcium-dependent pathway in cells where apoptosis is mitigated due to overexpression of Bcl-2 [74].

Other studies show that ceramide suppresses mitochondrial respiratory chain activity and regulates mitochondrial permeability of the inner mitochondrial membrane [56] (Figure 2). Thus, the level of ceramide is tightly controlled. In contrast, LacCer can increase mitochondrial ROS generation [75]. In the mitochondria of streptozotocin-induced diabetic rats, two observations were made. First, although the activity of ceramide synthase was high, the level of ceramide in mitochondria was low. Second, the level of LacCer in these mitochondria was high, suggesting that the glycosylation pathway (Figure 1) in these diabetic mice was predominant. Herein, ceramide was utilized to synthesize LacCer, thus lowering ceramide level. This was accompanied with a suppression of respiration activity as well as decreased capacity to retain calcium [76]. In another study using zebra fish and human macrophages, exposure to *Mycobacterium tuberculosis* led to the release of TNF-α, which generated mitochondrial ROS that activated lysosomal acid sphingomyelinase to generate ceramide and thus activate Bcl-2-associated X protein (BAX) [77]. In turn, BAX increased Ca^2+^ flow from the endoplasmic reticulum to the mitochondria by recruiting ryanodine receptors. The excess loading of Ca^2+^ into the mitochondria is believed to activate cyclophilin–induced necrosis [77]. Since mitochondria can glycosylate ceramide to LacCer, it may well participate in TNF-α–induced necrosis. This is supported by a report that showed increased host lipid metabolism in human tuberculosis granulomas [78].

In another study employing human MG-63 (wild-type, WT) osteosarcoma cells and a neutral sphingomyelinase deficient cell line, WT cells were prone to TNF-α–, ceramide–, and LacCer–induced apoptosis while the mutant cells were not [56]. However, when the mutant cells were fed ceramide and/or LacCer, it rendered these cells apoptotic. Furthermore, treatment with GSL synthesis inhibitor D-PDMP prior to incubation with ceramide but not LacCer mitigated apoptosis. Although treatment with D-PDMP raised the level of ceramide, it did not induce apoptosis in these cells as the activity of GlcCer synthase and LacCer synthase were blocked by D-PDMP. These findings suggest that the mutant cells utilized ceramide to generate LacCer to induce apoptosis. In WT and mutant cells, LacCer induced NADPH oxidase via increased expression of p92 phox and p47 phox to generate ROS [56].

Thus, ceramide generation shown in N-SMase-deficient mutant cells, mitochondria of diabetic mice, and human macrophages may not be sufficient to induce apoptosis and/or necrosis, respectively. Rather, the utilization of ceramide to synthesize LacCer may be required in these phenotypes. Thus, there are multiple ways by which LacCer could contribute to mitochondrial dysfunction and apoptosis/necrosis [56].

## 11. LacCer-Centric Pathways Leading to Inflammation

The inflammatory pathway involving LacCer has been proposed to be central to explaining the pathophysiology of atherosclerosis and potentially other inflammatory diseases. There are two major pathways explaining how LacCer contributes to inflammation: (i) the cell adhesion/migration pathway and (ii) the phospholipase A2 pathway.

**(i)**  **LacCer mediates a signaling pathway to induce cell adhesion/migration and inflammation**

Several studies in animal models of atherosclerosis and inflammation suggest that bacterial lipopolysaccharides induce the secretion of pro-inflammatory TNF-α. Moreover, ox-LDL is strongly associated with the pathogenesis of atherosclerosis and its uptake via macrophages results in the release of TNF-α [79]. In turn, TNF-α binds to its receptors on endothelial cells to activate a signaling pathway leading to the increased expression of several cell adhesion molecules—endothelial leukocyte cell adhesion molecule-1 (ELAM-1), vascular cell adhesion molecule (VCAM-1), and intercellular cell adhesion molecule-1 (ICAM-1)—on the surface of these cells (Figure 2) [80]. These cell adhesion molecules serve as ligands for other adhesion molecules, such as Mac-1 (CD11/CD18), expressed on circulating monocytes, neutrophils, lymphocytes, and eosinophils. Most importantly, ICAM-1 and VCAM-1 are expressed in human atherosclerotic plaques. In particular, ICAM-1 serves as a specific ligand for CD11/CD18 and lymphocyte function-associated antigen-1 (LFA-1) expressed on the surfaces of neutrophils and monocytes. Thus, adhesion of neutrophils and monocytes is mediated by cell adhesion molecules, allowing their trans-endothelial migration into the sub-endothelial space (Figure 2) and thereby contributing to inflammation and atherosclerosis (Figure 3). 

A head-to-head study in human endothelial cells treated with TNF-α and LacCer revealed that while TNF-α increased the expression of ICAM-1, VCAM-1, and ELAM-1, LacCer specifically increased the expression of ICAM-1 [81] at both the transcriptional and the translational levels. LacCer induced NADPH oxidase activation, and ROS generation was essential to ICAM-1 expression as overexpression of endogenous CuZn-superoxide dismutase via an adenoviral vector inhibited LacCer–induced ICAM-1 expression (Figure 2). In addition, in human neutrophils and monocytes, LacCer induced the expression of CD-11/CD18 [82], facilitating their adhesion to the endothelium and subsequent entry into the endothelium. This is considered the first step in inflammation and in atherosclerosis. Herein, in the sub-endothelial space, monocytes differentiate into macrophages. Macrophages express several scavenger receptors, the major ones being lectin-like ox-LDL (LOX-1), cluster of differentiation 36 (CD-36), and scavenger receptor-A-1 (SRA-1), which take up ox-LDL trapped in the extracellular matrix and ox-LDL circulating in the blood to form foam cells. Thus, these studies underscore the fundamental aspects of the initiation of inflammation and atherosclerotic plaque formation by way of adhesion of blood monocytes and neutrophils to the vascular wall endothelium and subsequent TEM (Figure 2) [83,84]. 

An exciting development linking TNF-α to LacCer and the inflammatory pathway involving cell–cell adhesion molecules was demonstrated as TNF-α was shown to dose-dependently increase the activity of LacCer synthase [81]. On the other hand, in human endothelial cells, blocking LacCer synthase activity with the use of D-PDMP completely mitigated TNF-α–induced ICAM-1 expression and adhesion to neutrophils. In sum, for the first time, this LacCer-centric pathway connects pro-inflammatory cytokine TNF-α, which targets LacCer synthase, to the initial step of inflammation and atherosclerosis. This pathway is found to be commonly shared in other biological systems and leads to diverse phenotypic endpoints including neuro-inflammation, inflammatory bowel disease, ischemia reperfusion injury, autophagy, and skin inflammation (see below).

**a.**  LacCer neuro-inflammation and inflammatory bowel disease

A potential role of LacCer synthase/LacCer in TNF-α–induced inflammatory response was recapitulated using rat primary astrocytes [85]. Upon stimulation with TNF-α, astrocytes expressed increased LacCer synthase activity and cell proliferation. This was mitigated with the use of D-PDMP as well as a 20-mer antisense oligonucleotide targeted against rat LacCer synthase. This led to decreased PI-3 kinase, Ras, and ERK1/2 expression as well as inhibiting astrocyte proliferation [39,85]. This inhibition was released with the addition of exogenous LacCer to the astrocyte culture, but not other glycolipids like glucosylceramide, glucocerebroside, GD1, GM1, and GM3 [39]. LacCer also activates inducible nitric oxide synthase enzyme, increasing nitric oxide production. Nitric oxide, normally a neuronal messenger, can become toxic at high concentrations and exacerbate neurodegenerative disorders like Alzheimer’s disease and multiple sclerosis. Furthermore, in an animal model of spinal cord injury, treatment with D-PDMP mitigated ERK1/2 activation, astrocyte proliferation, and improved recovery in rat post-spinal cord injury [86]. Additional evidence supporting the role of LacCer synthase/LacCer in proliferation, inflammation, and cancer was provided wherein transfection with antisense β-1,4GalT-V complementary DNA (cDNA) in cancer cells suppressed tumor development in animals [87]. 

LacCer has been implicated in inflammatory bowel disease, which include Crohn’s disease (CD) and ulcerative colitis (UC). While CD causes inflammation throughout the intestine tract, UC is typically restricted to the inner lining of the lower intestine or the colon. Further damage to the lining—sloughing off the cells, swelling and continued inflammation—collectively contributes to ulcerative colitis. A recent study using liquid chromatography with tandem mass spectroscopy (LC-MS) or quadrupole time-of-flight LC/MS (LC-QTOFMS) comparing the GSL composition of visibly normal versus ulcerative colitis tissues in patients revealed the following: the de novo biosynthesis of GSLs was reduced while LacCer synthesis was increased [88]. This observation was manifested in blood samples in ulcerative colitis patients. The association between LacCer and CD was first noticed in a scanning densitometry study that detected a strong LacCer signal in biopsies from patients with CD; no LacCer was detected in the unaffected parts of the bowels [89]. In a preliminary study with untargeted metabolomic analysis, the researchers noticed increased detection of LacCer in children with CD compared to the controls [90]. This observation was confirmed with a subsequent study where sphingolipid levels from healthy children and children with either CD or UC were quantified with high-performance LC-MS [91]. There was a significant increase in serum LacCer in children with CD and UC compared to controls. Neutrophils that invade the inflamed mucosa of CD and UC patients may be the source of LacCer. As patients with inflammatory bowel disease have a greater risk of developing colorectal cancer and LacCer and β-1,4GalT-V are elevated in colon cancer tissues, LacCer may be an important biomarker for these patients.

**b.**  LacCer, shear stress, and ischemia reperfusion injury

Since endothelial cells are exposed to blood, these cells are subject to hemodynamic factors. In particular, laminar shear stress leads to the release of ROS such as superoxide radicals. In response to such an assault, endothelial cells express ICAM-1 to initiate cell–cell adhesion and inflammation. However, the role of GSLs in this process was not known until recently. In a study in human endothelial cells, exposure to fluid shear stress (20 dynes/cm^2^) activated LacCer synthase, LacCer, and superoxide generation, followed by a signaling pathway including Ras, GDP loading, c-jun N-terminal kinase (JNK), extracellular-signal-regulated kinase (ERK), nuclear factor kappa B (NF-κB), c-Jun, c-Fos, and activator protein 1 (AP-1) leading to ICAM-1 expression [92]. This pathway was mitigated by the use of D-PDMP. Thus, this study updated the previous model of mechano-transduction by introducing LacCer synthase and LacCer as critical players in shear stress-induced injury.

Ischemia—the lack of oxygen—can be followed by ischemia reperfusion (perfusion of blood) injury to tissue during surgery; in vivo and in vitro hypoxia and re-oxygenation involves NADPH oxidase–induced ROS generation. Irrespective of the tissue, three main features in ischemia reperfusion injury are: the involvement of mitochondria [93], lipid peroxidation [94], and a transient increase in ceramide levels. Ceramide can induce cytochrome release from the mitochondria causing apoptosis. Ceramide may well be converted to LacCer to impair vital mitochondrial function involving Ca^2+^ homeostasis and respiration. Since Rac1 inhibition protects against hypoxia/reoxygenation-induced lipid peroxidation in human vascular endothelial cells [93], ischemia reperfusion injury may involve LacCer–induced NADPH oxidase activation (via Rac-1 activation) and ROS generation.

**c.**  LacCer and autophagy

Ceramide accumulation and up-regulation has also been documented in chronic inflammatory lung injury and lung diseases, e.g., chronic obstructive pulmonary disease (COPD), [95,96,97], causing pulmonary cell apoptosis and emphysema-like disease in experimental animals. In addition, studies show that exposure to cigarette smoke can induce inflammation/oxidative stress–induced emphysema in COPD [98]. Increased level of LacCer accompanied with increased expression of p62 in lungs from patients with COPD points to a role of this GSL in aberrant autophagy, thus contributing to the pathophysiology in cigarette smoke-induced emphysema. This tenet is substantiated further in bronchial epithelial cells and macrophages wherein exposure to an extract of cigarette smoke-induced LacCer synthesis, autophagy, and apoptosis [99] (Figure 2). Accordingly, treatment with D-PDMP, which blocks LacCer synthesis, decreased p62 expression, thus reversing cigarette smoke-induced COPD emphysema [99]. P62 is a protein which recognizes toxic cellular waste. Subsequently, the waste is removed by self-eating or autophagy. The accumulation of p62 is suggestive of dysfunctional autophagy-inducing stress and disease.

**d.**  LacCer and skin inflammation

The mammalian skin lipid matrix is endowed with high levels of ceramide. In particular, the long-chain fatty acid molecular species of ceramide in the skin have been accorded a vital role in hydration homeostasis [100,101]. Skin covers the entire human body and accounts for ~16% of total body weight. It provides a “biological carpet” for the growth of hair as well its coloration due to melanocyte function. While human aging is accompanied with greying of hair and balding in the vast majority of the global population, these phenotypes are accelerated upon the intake of a western diet and accompanied with several hematosed skin lesions. Interestingly, *ApoE^−/−^* mice fed a western diet display a similar pattern of hair loss and hair greying to that in man [102]. The *ApoE* protein serves as a ligand for receptors that transport and clear cholesterol. Ablation of this gene in mice renders them dyslipidemic, indicated by the presence of lipid deposits in peripheral tissues including the liver, lung, colon, and skin [103]. Thus, *ApoE^−/−^* mice are a suitable model to examine the role of GSLs in these phenotypes. On a regular chow diet, *ApoE^−/−^* mice grow lush black hair. However, chronic feeding of a western diet rich in fat and cholesterol for 8 weeks (from 12 weeks of age to 20 weeks) results in greying of hair and patches of hairless skin [102], resembling patients with decreased hair production also known as alopecia. Upon continuation of feeding western diet up to the age of 36 weeks, nearly 80% of the mouse body becomes devoid of hair and several hemotosed skin patches appear [102].

Chronic feeding of a western diet sets into motion a pro-inflammatory environment by increasing the levels of TNF-α inducible factor (TSG-6) and TNF-α and neutrophil migration (Figure 2). LacCer levels are also increased in the skin of *ApoE^−/−^* mice fed a western diet due to endogenous synthesis via activation of LacCer synthase by TNF-α and ox-LDL. The newly synthesized LacCer activates the cPLA2 pathway to cause inflammation via production of eicosanoids and cell adhesion molecules as well as trans-endothelial migration of neutrophils and monocytes. Hyperlipidemia in these mice also raises the serum level of ox-LDL, which is consumed by the monocytes to form fatty streaks or plaques. The net result of a western diet and the consequent double-prong attack by ox-LDL and TNF-α is a marked diminution of skin ceramide levels, thus contributing to skin lesion formation, hair loss, and hair discoloration. In addition, studies show that inactivation of Notch1, Notch2, or RBP-Jk (recombination signal binding protein for immunoglobulin kappa j region) in melanocytes can disturb the Notch signaling pathway and result in hair greying [104,105,106]. Since β-1,4GalT-V may also post-transcriptionally galactosylate Notch, it may well contribute to greying of hair. Thus, a western diet raises a highly oxidative stressful environment involving LacCer synthase activation and ROS generation causing LDL oxidation in a cyclical manner. This cycle can be broken by inhibiting LacCer synthase. Oral delivery of D-PDMP to *ApoE^−/−^* mice fed a chronic western diet can dose-dependently reverse hair discoloration, hair growth, and skin lesion formation [102].

**(iii)**  **LacCer recruits cytosolic phospholipase A2 pathway to induce cell adhesion and inflammation**

Activation of cytosolic phospholipase A2 by LacCer is a common feature in human endothelial cells and human neutrophils. Thereafter, the signaling pathways diverge to yield different phenotypes: (a) inflammation and cell adhesion in neutrophils and monocytes (Figure 2) and (b) angiogenesis in endothelial cells.

**a.**  LacCer and phospholipase A2 in inflammation and cell adhesion in neutrophils and monocytes

Neutrophils are blood cells that play a vital role in innate immune functions and inflammation. Accordingly, neutrophil infiltration into a tissue may be a hallmark in inflammation. LacCer is the predominant species of GSL in mature human neutrophils and contributes to nearly 70% of total GSLs. In neutrophils, LacCer is localized to the cell membrane [20] as well to the cytoplasm [107]. Exposure to exogenously supplied LacCer activates diverse signaling pathways in neutrophils. LacCer activates cPLA2 to cleave arachidonic acid from the sn-2 position in phosphatidylcholine, a precursor to the family of eicosanoids—prostaglandins, thromboxanes and leukotrienes—implicated in inflammation. In neutrophils, this is accompanied with the increased expression of CD11B/CD18, which can be mitigated by anti-CD18 and disparate PLA2 inhibitors. LacCer also activates NADPH oxidase which generates ROS. Superoxides generated as a consequence of LacCer-induced activation of NADPH oxidase has been suggested to facilitate neutrophil migration [108]. However, cPLA2 activation and not ROS generation is the upstream signaling molecule for CD11B expression, followed by neutrophil adhesion to ICAM-1 expressed in endothelial cells [82]. The activity of cPLA2α increases in response to high intracellular free calcium concentrations and phosphorylation by several kinases including ERK1/2 [109]. The binding of calcium to the C2 domain of cPLA2α stimulates the translocation of the enzyme to areas surrounding the nucleus. Once the enzyme simultaneously attaches to substrate membranes, cPLA2α can release arachidonic acid. 

However, recent studies show that certain lipids such as ceramide-1-phosphate and LacCer can activate cPLA2α by stimulating or promoting its attachment to substrate membranes in a calcium-independent manner [109]. In mouse L cells, treatment with TNF-α led to the generation of LacCer, the translocation of cPLA2 from the cytosol to the Golgi apparatus (a Ca^2+^-independent process/activation), and the release of arachidonic acid [110]. In another study, mouse fibroblasts cells treated with LacCer alone could simultaneously initiate phosphorylation of cPLA2α via the PKC/MEK/ERK-dependent pathway at the plasma membrane as well as the attachment of cPLA2α to substrate membranes such as the Golgi complex [109]. Similarly, in the CHO cell line, LacCer treatment resulted in a phosphorylation cascade and release of arachidonic acid [110]. Thus, LacCer appears to act as a phosphorylation initiator and anchoring molecule to activate cPLA2α. 

Studies show that in human monocytes (U-937), LacCer specifically activated protein kinase C (α and ε), which is translocated from the cytosol to the cell membrane. In turn, it activated calcium-independent PLA2 and/or cPLA2 to increase the expression of NF-κB (a heterodimer consisting of p65/RelA and p50/NF-κB1) by prostaglandin E2 (PGE2). PGE2 is the major prostaglandin generated in response to inflammation and it may induce NF-κB transactivation and increase expression of platelet-endothelial cell adhesion molecule (PECAM-1) [111] at both the transcriptional and translational levels. This is supported by the observation that a consensus sequence of NF-κB is associated with the promoter of the PECAM-1 gene [112]. However, LacCer did not increase the mRNA level of ICAM-1 in U-937 cells. Interestingly, in this study, the inhibition of endogenous LacCer synthesis with the use of D-PDMP did not affect PECAM-1 expression. This indicates that LacCer serves as an extracellular signaling molecule. This may be significant due to cell–cell exchange of GSLs.

**b.**  LacCer and VEGF-induced angiogenesis and inflammation in endothelial cells

Aberrant expression of vascular endothelial growth factor (VEGF) has been documented in diabetes, atherosclerosis, cancer metastasis, and inflammation, which contribute to the bulk of mortality and morbidity in man [113,114] (Figure 3). VEGF binds to its cognate receptors KDR (kinase insert domain receptor)/Flk-1, thus activating an elaborate signaling pathway leading to angiogenesis. Angiogenesis, the formation of new blood vessels from existing ones, is essential for fetal growth as well as tissue growth and repair and wound healing. However, uncontrolled angiogenesis is essential for tumor metastasis, diabetic retinopathy, and macular degeneration as well as several other serious human pathologies. Angiogenesis is a multi-process, multi-dimensional phenotype requiring a wide range of research to understand its mechanistic aspects. Developing and targeting novel pathways to control angiogenesis is an approach to prevent and mitigate these diseases. 

A major breakthrough in LacCer research and its relevance to angiogenesis-related diseases was made with a series of independent lines of inquiry demonstrating that VEGF–induced angiogenesis recruited LacCer synthase (β-1,4GalT-V) (Figure 2). These findings are the following: (1) In human umbilical vein endothelial cells and human arterial endothelial cells (HAEC), VEGF and β-fibroblast growth factor (β-FGF) recruited β-1,4GalT-V and LacCer to induce angiogenesis [115,116]. (2) Gene ablation of β-1,4GalT-V in cultured human umbilical vein endothelial cells renders these cells unresponsive to VEGF–induced angiogenesis [115]. (3) Pre-treatment of cells with several GSL glycosyltransferase inhibitors mitigated VEGF–induced angiogenesis to various degrees [115]. This was bypassed by treatment with LacCer, but not by other GSLs. (4) Conversely, treatment with L-PDMP, which activated LacCer synthase activity and cell proliferation, increased angiogenesis [50]. (5) Angiogenesis required LacCer–induced activation of PKC and cPLA2 as well as PECAM-1 expression, as treatment with PKC and PLA2 inhibitors and PECAM-1 monoclonal antibody blocked VEGF–induced angiogenesis. (6) Moreover, in vitro and in vivo experiments support a positive correlation between LacCer and cell proliferation, adhesion, invasion, and angiogenesis—all vital processes of carcinogenesis. In endothelial cells isolated from human colorectal cancer tissue, there was a 5-fold increase of β-1,4GalT-V mRNA transcripts but not β-1,4GalT-VI mRNA transcripts [116,117]. (7) Moreover, in a human colorectal cancer cell line (HCT-116), there was a cell density–dependent increase in the gene/protein expression of β-1,4GalT-V, LacCer mass, and cell proliferation. This was concomitantly mitigated by treatment with D-PDMP [45]. (8) On the other hand, overexpression of β-1,4GalT-V increased glioma cells [118]. Overexpression of β-1,4GalT-V gene in a glial cell line SHG44 transformed their morphology, increased invasiveness and migration in a matrigel matrix. Moreover, transfection with these cells increased tumor size ~10-fold. Conversely, treatment with short-hairpin RNA (shRNA) for β-1,4GalT-V decreased tumor volume [119]. LacCer may activate pro-survival pathways such as ERK1/2 and NK-κB signaling in cancer through increasing ROS generation [31]. Collectively, these studies unequivocally illustrate that β-1,4GalT-V gene and LacCer functioned as growth factors in glioma cancer cells.

The in vivo relevance of these observations became evident when oral delivery of D-PDMP, a GSL synthesis inhibitor, to nude mice markedly reduced (*p* < 0.005) VEGF–/β-FGF–induced angiogenesis concordant with diminished LacCer synthase activity [116]. Moreover, in a mouse xenograft model of renal cancer, a 30-fold increase in tumor volume was noted over of period of 26 days. This was accompanied with a 32-fold increase in LacCer in the kidney and a 2.5-fold increase in LacCer synthase activity. Oral delivery of D-PDMP markedly reduced tumor volume by 50% and significantly decreased LacCer synthase activity and mass. Unexpectedly, treatment with D-PDMP did not decrease the level of GlcCer; although it did significantly reduce GlcCer synthase activity in mice kidney compared to placebo [13]. 

Enzymes important in sphingolipid metabolism are deregulated in human colorectal tumors, human colon cancer cultures, and in rodent models of colon cancer. The altered metabolism is associated with an increased sphingosine-1-phosphate to ceramide ratio, which is linked to faster colon cancer progression [31]. In another study, the mRNA levels of β-1,4GalT-V was specifically increased in human colorectal cancer, contributing to tumor growth and metastasis. In human colorectal cancer tissue, there was also an upregulation of both β-1,4GalT-V activity and mass [45] as well as a most noticeable increase in the level of LacCer. Transfection of the antisense β-1,4GalT-V cDNA into certain cancer cells has been shown to suppress tumor growth in animals [87,120]. These earlier reports have been confirmed recently by the D-PDMP mediated downstream suppression of E2F1 (E2 transcription factor 1), a transcriptional regulator of cyclin D1 and D3 implicated in oncogenic transformation, and β-1,4GalT-V by RNA interference. This decreased tumorigenicity in glioma-initiating cells [121]. 

Another important observation emerging from additional studies suggests that LacCer alone is an angiogenesis inducer. For example, VEGF–induced angiogenesis requires VEGF to bind to the VEGF receptor tyrosine kinase [116]. This phenotype was mitigated with the use of SU-1498, a specific inhibitor of this tyrosine kinase, but was bypassed by LacCer. Second, feeding LacCer, but not GlcCer, digalactosyl-diglyceride, or ceramide, to human endothelial cells dose-dependently increased PECAM-1 expression and angiogenesis. Third, VEGF–induced angiogenesis was mitigated by dimethyl-sphingosine and suramin, two compounds known to inhibit sphingosine kinase and sphingosine-1-phosphate receptor, respectively [116]. This was bypassed by LacCer but not sphingosine-1-phosphate. VEGF post-transcriptionally activates PI-3 kinase required for the expression of PECAM1/CD36, an integral protein in human endothelial cells implicated in angiogenesis and the trans-endothelial migration of monocytes. VEGF–induced angiogenesis and trans-endothelial cell migration were both blocked with a PI-3 kinase inhibitor (LY 294002), Nw-nitro-L-arginine methyl ester (L-NAME, an inhibitor of nitric oxide synthase), 1-pyrrolodinecarbodithio acid (PDTC, an inhibitor of NF-κB), and PECAM-1 antibody [116]. Collectively, these reports suggest that PI-3 kinase activation and nitric oxide synthase activation are common features between VEGF and LacCer induced PECAM-1 expression and angiogenesis. In human arterial endothelial cells and in human umbilical vein endothelial cells, LacCer independent of VEGF can induce angiogenesis and the trans-endothelial migration of neutrophils and monocytes. When the LacCer synthase gene was knocked out in endothelial cells, PECAM-1 expression and angiogenesis decreased [122].

## 12. Conclusions 

The past few decades have witnessed a marked advancement in the development of mass spectrometry technology to quantify lipids, the availability of mouse models of human disease and pathology, and several molecular tools to dissect signaling pathways. These developments have led to numerous pre-clinical studies that help us better understand the interplay of GSLs in vascular biology and in the pathophysiology of diseases in order to develop novel drug targets and biomarkers of diseases. Thus, the major conclusions drawn in the field of LacCer metabolism are the following (Figure 3):

1. The LacCer-centric inflammatory pathway can begin to explain the pathophysiology of skin inflammation, neuro-inflammation, and hair greying/loss due to aging as well as other inflammatory diseases, e.g., COPD and inflammatory bowel disease.
2. A western diet, which induced skin inflammation, hair loss, and hair discoloration, implicates the LacCer biosynthetic pathway involving neutrophil infiltration and TSG-6 expression. This was reversed by blocking LacCer synthesis and raising skin ceramide levels.
3. TNF-α, a major pro-inflammatory cytokine, recruits players in the LacCer-centric inflammatory pathway, such as cPLA2 enzyme to generate eicosanoids/prostaglandins, leading to inflammation, neutrophil migration/infiltration, and expression of neutrophil/monocyte cell adhesion molecule, Mac-1 (CD11b). This LacCer-centric inflammatory pathway is shared in neuro-inflammation, ulcerative colitis, autophagy in emphysema due to COPD and cigarette smoking.
4. The LacCer-centric oxidative stress pathway may increase our understanding of atherosclerosis and lupus erythematosus via downstream generation of ROS to: (a) oxidize LDL in a cyclical fashion, raising the blood levels of oxidized LDL, (b) induce cell proliferation via activating phosphokinases Akt-1 and mTOR-C1 signaling cascade, (c) regulate cell–cell adhesion involving cell adhesion molecules, ICAM-1, PECAM-1, and Mac-1 (CD11b), (d) cause shear stress induced mechano-transduction and ischemia reperfusion injury, and (e) activate the cPLA2 enzyme and PECAM-1 gene expression to induce angiogenesis required for atherosclerotic plaque survival and cancer metastasis and tumor growth in colorectal cancer and possibly other types of cancer.
5. LacCer synthase is recruited and activated by VEGF and β-FGF to induce angiogenesis in vitro and in vivo via increased expression of ICAM-1 and PECAM-1 followed by monocyte and neutrophil TEM contributing to inflammation.
6. LacCer plays an important role in mitochondrial function by downregulating respiration and Ca^2+^ retention in diabetic rats. Both in mitochondria of diabetic mice and in N-SMase-deficient cells, ceramide generation may not be sufficient to induce apoptosis and necrosis; its glycosylation to LacCer is required.

In sum, LacCer synthase is a target for the convergence of diverse agonists that activate this enzyme to generate LacCer. In turn, LacCer activates NADPH oxidase to present an “oxidative stress” environment leading to several phenotypes in vivo and in vitro. LacCer can also activate the inflammatory pathway by activating cPLA2 and a cascade of other molecules. Both these pathways can be reversed or controlled by blocking LacCer synthase with the use of gene manipulation, glycosyltransferase inhibitors, and/or other therapies.

Perspectives

1. We are only beginning to understand the role of LacCer synthase in other inflammatory diseases such as Lupus Erythematosus. As Lupus predominantly afflicts women in their early reproductive years, this commands greater and urgent attention.
2. In diabetes, the mitochondria—the powerhouse of energy production and Ca^2+^ metabolism—becomes dysfunctional presumably due to increased LacCer levels. Further studies, including the targeting of drugs to improve mitochondrial health, are needed in this area. Such studies, in turn, will improve cardiac health.
3. Since knowledge of GlcCer and LacCer levels has increased the predictive value of atherosclerosis and diabetes disease onset and progression and cardiac function, these measurements could be prescribed as routine tests in clinical settings.
4. The pre-clinical studies above have provided a wealth of information on the role of LacCer synthase in several inflammatory diseases, cancer and atherosclerosis to set the stage for future human trials using various therapeutic modalities.

## Abbreviations

β-1,4 GalT-V	β-1,4 Galactosyltransferases V
β-1,4 GalT-VI	β-1,4 Galactosyltransferases VI
β-FGF	β-fibroblast growth factor
AP-1ApoE	Activator protein 1Apolipoprotein E
AS	Arterial stiffness
BAX	Bcl-2-associated X protein
Cer	Ceramide
CD	Crohn’s disease
CD-36	Cluster of differentiation 36
cDNA	Complementary DNA
CHO	Chinese hamster ovary
CoA	Coenzyme A
COPD	Chronic obstructive pulmonary disease
cPLA2	Cytosolic phospholipase A2
CVD	Cardiovascular disease
D-PDMP	D-threo-1-phenyl-2-decanoylamino-3-morpholino-1-propanol
E2FELAM	E2 transcription factorEndothelial leukocyte cell adhesion molecule
ER	Endoplasmic reticulum
ERKFH	Extracellular-signal-regulated kinaseFamilial hypercholesterolemic
HAEC	Human arterial endothelial cells
HCT	Human colorectal cancer
HDL	High-density lipoprotein
HIF-1A	Hypoxia-inducible factor 1-alpha
GbOse3Cer	Globotriosylceramide
GD3	Disialosyldihexosylceramide
GlcCer	Glucosylceramide
GM1	Monosialotetrahexosylceramide
GM3	Monosialodihexosylceramide
GSL	Glycosphingolipid
ICAM-1	Intercellular cell adhesion molecule-1
IL-23JNKKDR	Interleukin-23C-jun N-terminal kinase Kinase insert domain receptor
LacCer	Lactosylceramide
LC-MS	Liquid chromatography-mass spectrometry
LC-QTOFMS	Quadrupole Time-of-Flight liquid chromatography/mass spectrometry
LDL	Low-density lipoprotein
LFA-1	Lymphocyte function-associated antigen-1
LOX-1	Lectin-like ox-LDL
L-NAME	Nw-nitro-L-arginine methyl ester
L-PDMP	L-threo-1-phenyl-2-decanoylamino-3-morpholino-1-propanol
MAM	Mitochrondria-associated endoplasmic reticulum membrane
MAPK	Mitogen-activated protein kinase
MEK	Mitogen-activated protein kinase kinase
MIP-2	Macrophage inflammatory protein 2
MMP	Metalloproteinase
mRNA	messenger RNA
MS	Mass spectrometry
mTORC1	Mammalian target of rapamycin
NADH	Nicotinamide adenine dinucleotide hydride
NADPH	Nicotinamide adenine dinucleotide phosphate
Neu1	Nueraminidase-1
Neu3	Neuraminidase-3
NF-κB	Nuclear factor kappa B
N-SMase	Neutral sphingomyelinase
Ox-LDL	Oxidized low-density lipoprotein
PCNA	Proliferating cell nuclear antigen
PDGF	Platelet-derived growth factor
PDTC	Pyrrolodine-carbodithio acid
PECAM-1	Platelet-endothelial cell adhesion molecule-1
PGE2	Prostaglandin E2
PI-3	Phosphoinositide 3
PKC	Protein kinase C
PLA2	Phospholipase A2
POVPC	1-palmitoyl-2-(5-oxovaleroyl)-sn-glycero-3-phosphatidylcholine
PWV	Pulse-wave velocity
RBK-Jk	Recombination signal binding protein for immunoglobulin kappa j region
ROS	Reactive oxygen species
S1P	Sphingosine-1-phosphate
shRNA	Short hairpin ribonucleic acid
SOD	Superoxide dismutase
SRA-1	Scavenger receptor-A-1
TEM	Trans-endothelial migration
TNF-α	Tumor necrosis factor-alpha
TSG-6	TNF-α inducible factor
UC	Ulcerative colitis
UDP	Uridine diphosphate
VCAM-1	Vascular cell adhesion molecule-1
VEGF	Vascular endothelial growth factor
VLDL	Very low-density lipoprotein
WT	Wild-type
